# Cholangiocarcinoma Insights: Established Foundations and Cutting-Edge Innovations from Dr. James Cleary’s Pioneering Research

**DOI:** 10.3390/cancers16030632

**Published:** 2024-02-01

**Authors:** Viviana Cortiana, Harshal Chorya, Muskan Joshi, Shreevikaa Kannan, Diksha Mahendru, Harshitha Vallabhaneni, Helena S. Coloma, Yan Leyfman, Chandler H. Park

**Affiliations:** 1 Department of Medical and Surgical Sciences (DIMEC), University of Bologna, 40126 Bologna, Italy; 2 Medical College Baroda, Vadodara 390018, India; harshalchorya548@gmail.com; 3 Tbilisi State Medical University, Tbilisi 0186, Georgia; muskanj916@gmail.com (M.J.); shreevikaakkgs@gmail.com (S.K.); 4 Global Remote Research Scholars Program, St. Paul, MN 55101, USA; dikshamahendru02@gmail.com; 5 Apollo Institute of Medical Sciences and Research, Hyderabad 517001, India; harshithabangaaru@gmail.com; 6 Harvard University, Cambridge, MA 02138, USA; helenaisabellacoloma@gmail.com; 7 Icahn School of Medicine at Mount Sinai, New York, NY 10029, USA; yan.leyfman@mssm.edu; 8 Norton Cancer Institute, Louisville, KY 40202, USA; chandler.park@louisville.edu

**Keywords:** cholangiocarcinoma, biliary tract cancers, fibroblast growth factor receptor (FGFR), IDH (isocitrate dehydrogenase), insertions and deletions (INDELs)

## Abstract

**Simple Summary:**

Biliary tract malignancies, including cholangiocarcinoma (CCA) and gallbladder neoplasms, present intricate challenges in diagnosis and treatment. This comprehensive overview highlights the diversity of CCA subtypes, their genetic underpinnings, and the pivotal role of etiological factors. Despite diagnostic complexities, advancements in genomic sequencing reveal promising therapeutic targets, such as FGFR2 alterations and IDH1 mutations. Dr. James Cleary’s insights underscore the potential of these targets in reshaping the treatment landscape. This paper provides insights into the conventional understanding of biliary tract malignancies and emphasizes the critical need for ongoing research to optimize outcomes in this challenging cancer subtype. By elucidating diverse FGFR2 alterations and the potential of IDH1 inhibition, the aim is to pave the way for targeted therapeutic interventions. As we delve deeper into understanding the intricate complexities of biliary tract malignancies, this research seeks to drive precision medicine forward, offering hope for improved treatment strategies and outcomes in the face of this formidable cancer.

**Abstract:**

This paper provides insights into the conventional understanding of biliary tract malignancies, with a specific focus on cholangiocarcinoma (CCA). We then delve into the groundbreaking ideas presented by Dr. James Cleary. CCA, originating from biliary tree cells, manifests diverse subtypes contingent upon anatomical localization and differentiation status. These variants exhibit discrete genetic aberrations, yielding disparate clinical phenotypes and therapeutic modalities. Intrahepatic, perihilar, and distal CCAs intricately involve distinct segments of the biliary tree, further categorized as well-differentiated, moderately differentiated, or poorly differentiated adenocarcinomas based on their histological differentiation. Understanding the etiological factors contributing to CCA development assumes paramount importance. Stratifying these factors into two groups, those unrelated to fluke infestations (e.g., viral hepatitis and fatty liver conditions) and those associated with fluke infestations (e.g., chronic liver inflammation), facilitates predictive modeling. The epidemiology of CCA exhibits global variability, with Southeast Asia notably displaying higher incidences attributed primarily to liver fluke infestations. Jaundice resulting from bile duct obstruction constitutes a prevalent clinical manifestation of CCA, alongside symptoms like malaise, weight loss, and abdominal pain. Diagnostic challenges arise due to the symptomatic overlap with other biliary disorders. Employing comprehensive liver function tests and imaging modalities such as computed tomography assumes a pivotal role in ensuring accurate diagnosis and staging. However, the definitive confirmation of CCA necessitates a biopsy. Treatment modalities, predominantly encompassing surgical resection and radiation therapy, hold curative potential, although a considerable subset of patients is deemed unresectable upon exploration. Challenges intensify, particularly in cases classified as cancer of unknown origin, underscoring the imperative for early intervention. Advancements in genomic sequencing have revolutionized precision medicine in CCA. Distinct genomic markers, including fibroblast growth factor receptor 2 (FGFR2) alterations and isocitrate dehydrogenase 1 (IDH1) mutations, have emerged as promising therapeutic targets. FGFR2 alterations, encompassing mutations and rearrangements, play pivotal roles in oncogenesis, with FGFR inhibitors demonstrating promise despite identified resistance mechanisms. Similarly, IDH1 inhibitors face challenges with resistance, despite encouraging early clinical trial results, prompting exploration of novel irreversible inhibitors. Dr. James Cleary’s illuminating discourse underscores the significance of diverse FGFR2 alterations and the potential of IDH1 inhibition in reshaping the treatment landscape for CCA. These findings unveil critical avenues for targeted therapeutic interventions, emphasizing the critical need for ongoing research to optimize outcomes in this challenging cancer subtype, incorporating innovative insights from Dr. Cleary.

## 1. Introduction

**Figure 1:**
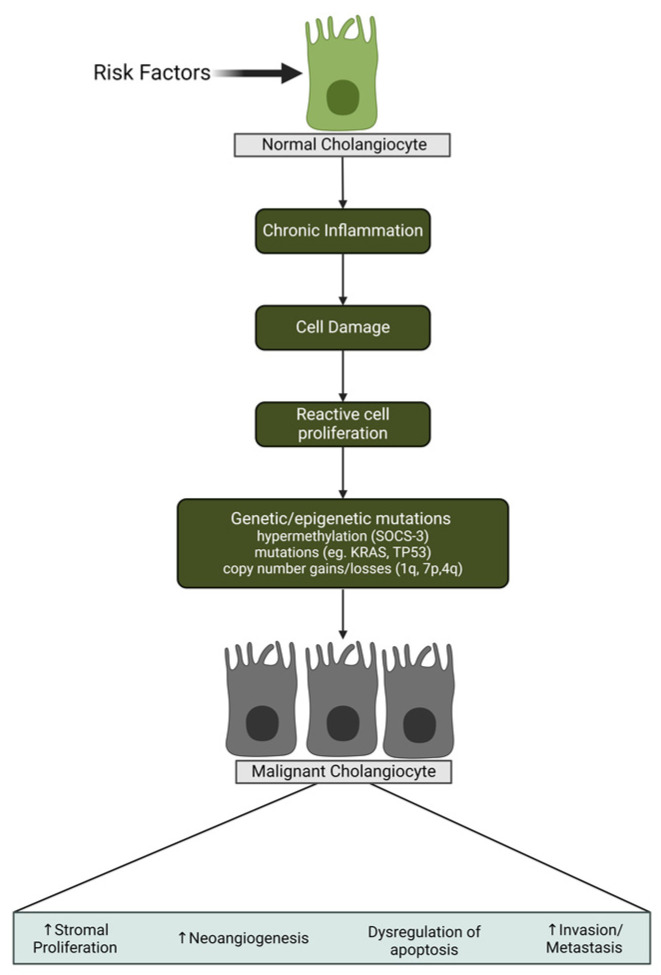
The figure in the paper succinctly outlines the molecular mechanisms behind cholangiocarcinoma development, featuring key elements such as genetic mutations (KRAS, TP53, BRAF), chronic inflammation’s role, epigenetic changes, and dysregulated signaling pathways [4].

Biliary tract cancers, encompassing cholangiocarcinoma (CCA) and gallbladder cancer, exhibit varied subtypes arising from different locations within the biliary tree with cholangiocyte differentiation [1]. Anatomical classifications, such as intrahepatic, perihilar, and distal CCA, are characterized by distinct genetic features, clinical presentations, and management options [2,3]. These are very aggressive cancers, and patients can get sick rapidly. Therefore, determining molecular mechanisms and risk factors that can lead to non-fluke and fluke-related CCA is the highest priority, integrating both established knowledge and innovative perspectives from Dr. Cleary. CCA development is a multifaceted process influenced by various molecular mechanisms. Genetic alterations, such as mutations in the KRAS, TP53, and BRAF genes, contribute to uncontrolled cell growth and survival. Chronic inflammation, particularly associated with conditions like Primary Sclerosing Cholangitis (PSC) or infection with liver flukes, creates a microenvironment favorable for tumor initiation. Epigenetic changes, including DNA methylation and histone modifications, play a role in regulating gene expression patterns. Dysregulation of signaling pathways, such as Notch, Wnt/β-catenin, and EGFR, influences cell fate determination and proliferation. The tumor microenvironment undergoes alterations, with changes in the extracellular matrix and the presence of cancer-associated fibroblasts impacting disease progression [4] (Figure 1).

Non-fluke-related cases, on the rise in the U.S., result from factors like primary sclerosing cholangitis and viral hepatitis. In contrast, liver flukes, parasites causing chronic inflammation, contribute to fluke-related cases. Global epidemiology reveals diverse incidence rates, with Southeast Asia, particularly Thailand, reporting higher numbers. In the U.S., there are approximately 10,000 cases annually, with an estimated incidence of 1.6 cases per 100,000 patients. Geographical variations persist, as seen in North America and Europe, where the American Cancer Society estimated around 14,480 new cases in 2022. Globally, in 2020, there were about 38,000 new cases of liver and intrahepatic bile duct cancers, with CCA representing a significant proportion. Regional disparities, linked to lifestyle, dietary habits, and risk factors, underscore the multifaceted nature of CCA epidemiology. Ethnic and racial disparities, including higher rates among Asian and Pacific Islander populations in the U.S., add complexity. Genomic differences between liver fluke-related cases in Thailand and non-fluke cases in the U.S. highlight the cancer’s heterogeneity. Understanding and treating CCA necessitates a multidisciplinary approach due to its complex nature, including genetic predispositions and geographic variations. In this commentary, we delve into the groundbreaking research of Dr. James Cleary, MD, Ph.D., a prominent medical oncologist and clinical investigator at the Hale Family Center for Pancreatic Cancer Research, affiliated with the Department of Medical Oncology at the Dana-Farber Cancer Institute. He specializes in treating gastrointestinal cancers and has played a pivotal role in advancing the understanding and management of these complex malignancies. This commentary focuses on synthesizing key insights from Dr. Cleary’s discourse, shedding light on his significant contributions to the treatment landscape, particularly in CCA.

## 2. Clinical Manifestations and Diagnostic Challenges in Cholangiocarcinoma: Unraveling the Complexities of Presentation and Diagnosis

Before delving into Dr. Cleary’s contributions, let us briefly summarize the conventional diagnostic approaches and clinical manifestations of CCA, a challenging cancer to diagnose, commonly presents with jaundice due to the obstruction of bile ducts by the tumor [1]. Beyond jaundice, patients often exhibit a spectrum of symptoms, including right upper quadrant abdominal pain, malaise, night sweats, asthenia, nausea, and weight loss [5]. The obstruction of bile flow by the tumor results in bile leakage into the bloodstream, ultimately leading to jaundice [1]. Various other factors, such as parasitic infection, biliary cysts, duodenal diverticula, and pancreatitis, can also contribute to impaired biliary drainage.

In the evaluation of suspected CCA, consideration of chronic hepatic dysfunctions is imperative. The Courvoisier-Terrier sign serves as a prominent characteristic for neoplastic lesions causing bile duct obstruction, a well-established diagnostic aspect, complemented by innovative diagnostic challenges discussed by Dr. Cleary. Before imaging, a comprehensive liver function test (LFT) blood panel is recommended [5].

Computed tomography stands as the cornerstone imaging modality for the assessment and staging of CCA. In cases involving cirrhotic livers, intrahepatic CCA (iCCA) often exhibits distinct imaging patterns, including arterial peripheral-rim enhancement progressing into uniform contrast uptake during delayed or stable phases across dynamic sequences. Notable features such as delayed enhancement, capsular retraction, vascular invasion, or the presence of satellite nodules strongly suggest the presence of iCCA. Clinical diagnosis is commonplace, with imaging and immunohistochemistry revealing markers like CK7 and CK19, complemented by techniques such as albumin in situ hybridization. Biopsy remains essential for the definitive confirmation of CCA diagnosis [5].

Commonly, early management strategies involve surgery [1]. Radical surgical resection stands as the potential curative approach for CCA; however, a significant proportion of patients (10–45%) are deemed unresectable during exploratory laparotomy [6]. CCA often presents as a challenging case of cancer of unknown primaries, complicating treatment decisions. Therefore, given the difficulty in identifying the primary site in some cases, when CCA is localized within the bile duct, it is often considered a priority in terms of diagnosis and treatment decisions, as localized tumors may be more amenable to specific treatments, and knowing the primary site can guide the healthcare team in making more informed decisions regarding the best course of action for the patient [1].

## 3. Advancements in the Management of Metastatic Cholangiocarcinoma: From Standard Chemotherapy to Emerging Precision Medicine Strategies

In this section, we distinguish between standard chemotherapy practices and the emerging strategies advocated by Dr. James Cleary in the management of metastatic CCA. Metastatic CCA, presenting a formidable challenge in treatment, primarily focuses on prolonging patient survival and enhancing their quality of life. Surgical resection is precluded in metastatic cases, necessitating a reliance on systemic chemotherapy. Historically, the gemcitabine/cisplatin regimen emerged as the standard first-line treatment, as evidenced by the ABC-02 study conducted across multiple English hospitals, demonstrating its superiority over single-agent gemcitabine in advanced biliary cancer (ABC). The definition ABC refers to malignancies that have reached an advanced stage of growth and may have spread beyond the primary site in the bile ducts. This term is often used to describe cancers, such as CCA, that exhibit extensive local invasion or metastasis to distant organs, making them more challenging to treat [7]. However, the absence of FDA-approved targeted therapies prompted a shift towards exploring novel avenues.

Dr. James Cleary’s enlightening discussion furthered this exploration by spotlighting a recent study that integrated immunotherapy (PD1 drug durvalumab) with gemcitabine/cisplatin, revealing improved outcomes. Despite these advancements, the modest benefits and demographic variations, particularly in Asian patients with liver fluke-associated CCA, raise questions about broader applicability [1,8]. As patients transition to second-line chemotherapy after the eventual failure of initial treatment, options remain limited. The ABC 06 group’s trial underscored FOLFOX as modestly superior to best supportive care, underscoring the pressing need for ongoing research [9]. The NIFTY trial introduced 5FU/Liposomal Irinotecan as a second-line option with a 3.1-month benefit, emphasizing the ongoing quest for diverse treatment approaches [10]. Recognizing the genomic diversity within CCA patients, the oncology community is steering toward precision medicine. Departing from the one-size-fits-all approach, there is a growing emphasis on tailoring treatments to specific molecular subgroups. Drawing inspiration from the success in lung cancer, where genomic profiling has revolutionized outcomes, the aim is to apply similar principles to CCA treatment.

In summary, the transition from conventional chemotherapy to precision medicine in the management of metastatic CCA represents a dynamic landscape of research and evolving strategies, integrating both established knowledge and Dr. Cleary’s innovative contributions, to enhance patient outcomes. Ongoing FDA considerations and continued research will shape the future of treatment protocols for this challenging condition, facilitating a seamless transition in the narrative of our paper.

## 4. Unraveling Genomic Diversity: Insights from Tumor Sequencing and Precision Medicine in Cholangiocarcinoma

Before delving into Dr. Cleary’s insights, let us elucidate the existing genomic markers and knowledge in CCA. The diverse genomic markers identified in CCA cancers have paved the way for targeted drug treatments tailored to specific genomic mutations [1]. Beyond providing prognostic insights, the mutational profile sheds light on therapeutic sensitivity [11]. Numerous genomic alterations associated with CCA, including but not limited to fibroblast growth factor receptor 2 (FGFR2), isocitrate dehydrogenase 1 and 2 (IDH1/2), ERBB2 or Catenin Beta 1 (CTNNB1), KRAS, NRAS, BRAF, mismatch-repair deficiency, HER2 (ERBB2), ALK, ROS1, or NTRK, PDGFRA, and RET, have been identified [11,12].

However, while this holds, particularly for intrahepatic CCA, data remain limited for biliary and extrahepatic cancers [1]. One of the most prevalent actionable immunohistochemistry (IHC) mutations is the FGFR alteration [1,13]. Upon protein dimerization activation, FGFR initiates cascades of signaling pathways, including PI3K-AKT or RAS-MAPK pathways, regulating tissue development and repair [14,15]. Various mutations in FGFR, such as amplifications, rearrangements, mutations, fusions, and indels, are implicated in carcinogenesis [12,16]. These mutations induce FGFR protein dimerization, leading to heightened signaling that prompts cell proliferation, angiogenesis, and immune escape [14].

While FGFR2 inhibitors exhibit efficacy, they are not without drawbacks. Nevertheless, the associated side effects are generally milder and better tolerated compared to chemotherapy. Common adverse effects include alopecia, diarrhea, fatigue, dysgeusia, nail changes with onycholysis, mucosal dryness, ocular toxicity, nausea, anorexia, diarrhea, and constipation [13,15]. Hyperphosphatemia, a frequent side effect, results from FGFR1 blockade regulating phosphorus excretion in renal tubules and can be effectively managed through a low-phosphate diet, phosphate binders, dose adjustment, or diuretics [1,15]. Resistance to FGFR inhibitors emerges in patients after a specific treatment duration, attributed to factors such as secondary FGFR kinase domain mutations or the development of FGFR2-independent mechanisms [16,17]. The gatekeeper mutation is a common alteration, altering the drug-binding site conformation and causing a steric clash [16]. Dr. James Cleary introduces a groundbreaking class of drugs in CCA treatment, termed Irreversible FGFR inhibitors. Despite mutations, these structurally modified drugs can bind and adapt to the FGFR ligand, offering hope for patients with CCA [18].

## 5. Exploring Alternative FGFR2 Alterations and the Promise of IDH1 Inhibition in Cholangiocarcinoma

Dr. James Cleary’s enlightening keynote presentation provided a deep dive into the exploration of alternative Fibroblast Growth Factor Receptor 2 (FGFR2) alterations and the potential therapeutic implications of Isocitrate dehydrogenase 1 (IDH1) inhibition [1]. Beyond the well-established translocations, attention was directed toward less common FGFR2 Insertions and Deletions (INDELs) located in the protein’s extracellular domain [19]. Notably, these INDELs, responsible for disrupting crucial regulatory protein portions, intrigued researchers due to their association with congenital syndromes like congenital craniocytosis [20,21]. Dr. Cleary underscored the significance of identifying and targeting these less frequent alterations, citing a case where successful treatment with an FGFR2 inhibitor persisted for over three years, even in the presence of resistance mutations [12]. Moreover, the discussion delved into the intriguing realm of IDH1 alterations in CCA, emphasizing the unique nature of mutated IDH1, which generates the oncometabolite 2-hydroxyglutarate, absent in normal cells [22,23]. This oncometabolite hinders cellular differentiation, contributing to cancer progression. The potential of targeting IDH1 mutations was highlighted, drawing parallels with successes in gliomas and Acute Myeloid Leukemia (AML) [24,25]. While IDH1 inhibitors like Ivosidenib demonstrated some success, acquired resistance, especially with secondary IDH1 mutations and mutations in Isocitrate dehydrogenase 2 (IDH2), posed challenges [26,27]. Dr. Cleary proposed a strategy targeting both IDH1 and IDH2 to combat resistance, envisioning a future where combination therapies could extend the efficacy of treatments. The discussion further addressed the growing resistance to Ivosidenib, prompting the exploration of novel irreversible IDH1 inhibitors, showing promise in preclinical studies [28]. The presentation stressed the potential of these inhibitors to overcome resistance mechanisms, incorporating both established knowledge and the rationale behind FGFR inhibitors, as well as Dr. Cleary’s insights into the promise of IDH1 inhibition.

In conclusion, Dr. Cleary’s comprehensive study illuminated the significance of diverse FGFR2 alterations and the evolving promise of IDH1 inhibition as a treatment for CCA. These insights offer crucial perspectives for therapeutic approaches, paving the way for more effective management strategies. Continued research is essential to optimize treatment outcomes for patients grappling with this complex cancer subtype.

## 6. Conclusions

In conclusion, this manuscript distinguishes between the well-established knowledge and the innovative ideas presented by Dr. James Cleary. His contributions inspire hope for more effective management strategies in the future. Dr. James Cleary’s groundbreaking research on CCA, particularly his focus on the novel subset with FGFR2 fusions, signifies a significant stride in targeted therapies. His leadership in clinical trials, such as the PARP inhibitor Niraparib in pancreatic cancer, showcases a commitment to translating scientific discoveries into impactful treatments [12,29,30]. The encouraging outcomes from the ClarIDHy trial, leading to an FDA application, underscore the potential clinical benefits of Dr. Cleary’s work, offering hope for improved outcomes in CCA patients.

Dr. Cleary’s keynote presentation offers profound insights into the dynamic landscape of CCA, highlighting its multifaceted challenges in clinical manifestation, diagnosis, and therapeutic management. The intricate nature of CCA demands a multidisciplinary approach, acknowledging various risk factors such as geographic variations and genetic predispositions. Despite notable advancements, exemplified by the groundbreaking Advanced Biliary Tract Cancer (ABC)-02 study, CCA remains a perplexing entity. Dr. Cleary’s elucidation serves as a guiding light, facilitating a deeper comprehension of its complexities. The potential discovery of novel therapeutic pathways, particularly through targeted agents like FGFR2 and IDH1 inhibitors, holds promise for transforming the management of this intricate malignancy in the future. Nevertheless, it is crucial to recognize prevailing challenges, notably the development of resistance to targeted treatments, which stands as a substantial obstacle. Researchers and clinicians are actively addressing these challenges, underscoring the imperative for ongoing exploration and innovation in the field. Looking forward, the medical community remains steadfast in unraveling the mysteries of CCA. Dr. Cleary’s contributions, coupled with continuous research efforts, inspire hope for the development of personalized approaches and efficient treatments, distinguishing between well-established practices and the innovative pathways illuminated by Dr. Cleary.

## Data Availability

No patient data were directly utilized in this study.
